# Temporal Distribution of Milking Events in a Dairy Herd with an Automatic Milking System

**DOI:** 10.3390/ani15152293

**Published:** 2025-08-06

**Authors:** Vanessa Lambrecht Szambelan, Marcos Busanello, Mariani Schmalz Lindorfer, Rômulo Batista Rodrigues, Juliana Sarubbi

**Affiliations:** 1 Department of Animal Science, Federal University of Santa Maria, Palmeira das Missões 98300-000, RS, Brazil; nessa.szambelan@gmail.com (V.L.S.); rrodrigues1903@gmail.com (R.B.R.); juliana.sarubbi@ufsm.br (J.S.); 2 Department of Animal Science, Federal University of Paraná, Curitiba 80035-050, PR, Brazil; 3 Department of Animal Science, College of Agriculture, “Luiz de Queiroz”/University of São Paulo—ESALQ/USP, Piracicaba 13418-900, SP, Brazil; 4 Department of Veterinary Sciences, Regional University of Northwestern Rio Grande do Sul—Unijuí, Ijuí 98700-000, RS, Brazil; mariani.lindorfer@delaval.com

**Keywords:** dairy cow behavior, voluntary milking, lactation stage, parity order, seasons

## Abstract

This study aimed to evaluate daily patterns of hourly milking frequency (MF) in dairy cows milked with an automatic milking system (AMSs), considering the effects of season, parity order (PO), days in milk (DIM), and milk yield (MY). A retrospective longitudinal study was conducted on a commercial dairy farm in southern Brazil over one year using data from 130 Holstein cows and 94,611 milking events. MF data were analyzed using general linear models. Overall, hourly MF followed a consistent daily pattern, with peaks between 4:00 and 11:00 a.m. and between 2:00 and 6:00 p.m., regardless of season, PO, DIM, or MY category. MF was higher in primiparous (2.84/day, *p* = 0.0013), early-lactation (<106 DIM; 3.00/day, *p* < 0.0001), and high-yielding cows (≥45 L/day; 3.09/day, *p* < 0.0001). High-yielding cows also showed sustained milking activity into the late nighttime. Although seasonal and individual factors significantly affected MF, they had limited influence on the overall daily distribution of milkings. These results suggest stable behavioral patterns within the specific AMS management conditions observed in this study and suggest that adjusting milking permissions and feeding strategies based on cow characteristics may improve system efficiency.

## Simple Summary

Automatic milking systems (AMSs) give cows the freedom to choose when to be milked, but their milking behavior often follows consistent patterns influenced by biological and management factors. In this study, we analyzed over 94,000 milking events from 130 cows over one year on a Brazilian dairy farm using AMSs. Milking frequency (MF) followed a clear daily pattern, with most milkings occurring in the early morning (4:00 to 11:00 a.m.) and afternoon (2:00 to 6:00 p.m.). Although this pattern remained consistent, its intensity varied according to individual and seasonal factors. Cows in early lactation (<106 days in milk) showed higher MF throughout the day, including during nighttime hours, averaging 3.00 milkings/day. Primiparous cows had the highest MF (2.84 milkings/day), with earlier morning activity compared to older cows. High-producing cows (≥45 L/day) maintained elevated MF from afternoon to night (2:00 p.m. up to 2:00 a.m.), averaging 3.09 milkings/day. These findings highlight that, while AMS offers voluntary access, cow behavior is still influenced by lactation stage, parity, and milk yield. Recognizing these patterns can help farmers optimize cow traffic, feeding strategies, and robot efficiency in AMS-managed herds.

## 1. Introduction

Automatic milking systems (AMSs) have seen growing adoption worldwide. Modern dairy operations are increasingly turning to automation to balance rising productivity demands with sustainable animal management practices. Numerous studies have aimed to understand the specific dynamics of these systems, addressing aspects such as cow traffic design [1], feeding strategies and management, milking permission settings [2,3], milking performance [4], and the occurrence of diseases like mastitis [5] and lameness [6]. Despite these advances, AMS remains a costly investment for dairy farmers, making efficient operation essential.

A key factor influencing AMS efficiency is milking frequency (MF), since more frequent milkings generally results in higher milk yield per robot per day. An AMS can perform between 150 and 180 milkings per day, considering 7 to 8 milkings per hour over approximately 22 h of daily availability [7]. Therefore, maximizing milking events is desirable to ensure optimal utilization and productivity of the system.

Research on MF in AMS has primarily focused on concentrate supply in the robot [2,8], feed delivery frequency and feed push-ups [9,10], and cow traffic designs that could affect voluntary milkings [11,12]. Conversely, the number of fetch cows, those that need to be actively fetched to the robot, is associated with reduced AMS efficiency [7,13]. Minimizing cow fetching and encouraging voluntary visits are crucial strategies for improving AMS performance, especially when labor availability for fetching is limited.

Unlike conventional milking systems, where cows are milked two or three times daily at fixed times, an AMS allows cows to establish their own milking routines. Studies suggest that MF and milking routines depend largely on individual cow behavior [14,15]. However, cows are naturally gregarious and tend to synchronize activities such as feeding. For instance, Pitkaranta et al. [13] reported that if a cow cannot feed while others are eating, she is unlikely to feed alone later. Oberschätzl-Kopp et al. [9] and Matson et al. [10] observed that milkings increase substantially after feed delivery and moderately after feed push-ups. Feed delivery and feed push-up stimulate cow movement and feeding behavior, which in turn encourage cows to visit the milking units more frequently, increasing MF.

In AMS, MF tends to be higher during daytime hours and lower at night, reflecting cows’ natural behavior of feeding more during the day and resting at night [15,16]. Despite this, detailed information on the specific hours when milking is most frequent in AMS remains limited. Therefore, this study aims to analyze the distribution of milking events throughout the day in an AMS herd located in southern Brazil. Additionally, we investigate whether factors such as season, parity order (PO), days in milk (DIM) period, and milk yield affect daily milking patterns. We hypothesize that these factors influence the temporal distribution of milking frequency, leading to distinct daily milking patterns among cows in AMS.

## 2. Materials and Methods

### 2.1. Study Design

This study employed a retrospective longitudinal design following the recommendations of the STROBE statement for reporting observational studies [17]. The study covered a one-year period from 22 May 2022 to 22 May 2023.

### 2.2. Farm Characteristics

The data were provided by a dairy farm from Eugênio de Castro County, Rio Grande do Sul State, Brazil, located at latitude 28°31′35″, longitude 54°8′50″, and an altitude of 312 m. The region has a Cfb-type climate with temperate summers [18].

In this dairy farm, lactating cows were maintained in a compost-bedded pack barn equipped with fans and sprinklers positioned along the feed alley and in the waiting area. Feeding was performed twice daily (at 6:00 a.m. and 4:30 p.m.) using a mechanical feed wagon to distribute a partial mixed ration. Feed push-ups were carried out throughout the day whenever necessary to ensure feed availability. Diet changes throughout the year occurred solely due to the availability and cost of ingredients in the region. The usual diet included corn silage, ground corn, wheat haylage, wheat straw, soybean meal, expeller soybean meal, mineral salt, a buffering agent, cottonseed, and soybean hulls. Diet was formulated according to NRC Dairy Cattle [19].

Cows were milked in two DeLaval^®^ AMS robotic units (V300 model, DeLaval International A.B., Tumba, Sweden) operating 24 h per day. Cows had unrestricted access to both AMS units and were free to choose their preferred milking station. The remaining feed (a concentrate feed) was supplied through the AMS units, with amounts adjusted individually based on each cow’s performance and DIM stage. A milk-first cow traffic design was used in this dairy farm. In this cow traffic design, cows are guided by a selection gate to the feeding area if they have no permission for milking or to the waiting area if they have permission for milking [1]. The selection gate is a unique way to access the feeding area, and when cows decide to lie down, they can access the lying area via one-way gates [1].

The AMS employed different milking permission settings that varied according to DIM stage, PO (primiparous or multiparous), and each cow’s performance. For milking permission, cows were categorized into three DIM stages: 1 to 110 days, more than 111 days, and less than 90 days before calving. Thus, three DIM categories were established to encompass the beginning, middle, and end of lactation. At the beginning of lactation, more permissions were granted to stimulate milk production. At the end of lactation, fewer permissions were given to facilitate the drying-off process.

Cows in early lactation (1 to 110 DIM) were allowed access to the milking robot after 5 h (primiparous) or 6.5 h (secundiparous and multiparous) from the previous milking, or if their expected milk yield exceeded 8 kg. In mid-lactation (DIM > 111 days), primiparous cows received permission after 6.5 h or if expected milk yield exceeded 9 kg, while multiparous cows were allowed after 7 h or if expected milk yield surpassed 10 kg. In late lactation (within 90 days before calving), primiparous cows were granted access after 9.5 h, and multiparous cows after 10 h, or in both cases if expected milk yield exceeded 10 kg. Cows are considered milk-delayed if not milked within a defined period after receiving permission: 8.5 h (DIM 1–110), 9.5 h (DIM > 111), and 12 h (within 90 days before calving), regardless of PO. Milk-delayed cows were red-flagged by the system, which monitors whether fetching them is required. On the farm, cows were manually fetched for milking at specific times based on labor availability: 05:00 a.m., 1:00 p.m., and 6:00 p.m. In addition, the AMSs undergo scheduled cleanings at 03:00 a.m. and 8:00 p.m., during which milking operations are temporarily paused for complete system sanitation.

During the study period, the farm maintained a monthly median of 100 lactating Holstein cows. The median was reported to account for fluctuations in herd size, which ranged from 80 to 130 cows milked daily in the AMS units. The dairy farm management conditions remained consistent throughout the study period.

### 2.3. Dataset Collection and Handling

In this dairy farm, data were collected daily by the AMS and stored in the DelPro^®^ software (version 10.7) (DeLaval International A.B., Tumba, Sweden). The records included each cow’s average daily milk yield, the time of each milking session, MF, and individual characteristics such as PO and DIM stage. The data were obtained from 130 individual cows milked in the AMS during the study period, comprising a total of 94,611 milking events.

The data were categorized for subsequent analysis. Parity order was classified as primiparous (first-calving cows), secundiparous (second-calving cows), and multiparous (third or greater calving cows). Days in milk were categorized as ≤105 DIM, 106–205 DIM, and >205 DIM, whereas milk yield categories (MYCat) were defined as <25 kg, ≥25 to <35 kg, ≥35 to <45 kg, and ≥45 kg.

### 2.4. Statistical Analysis

The MF data were analyzed using a general linear model to evaluate the effects of hour, season (or PO, DIM, and MYCat), and their interactions. The model used for this analysis is described below in Equation (1):

(1) $$y_{ijk}=\mu +E_{i}+H_{j}+{E\times H}_{\left(ij\right)}+\varepsilon_{ijk}$$

where

$y_{ijk}$ = hourly milking frequency;
μ = overall mean;
$E_{i}$ = effect of i-th level of season (i = 4), parity order (i = 3), days in milk (i = 3), or milk yield category (i = 4);
$H_{j}$ = effect of j-th hour, where j = 24;
${E\times H}_{\left(ij\right)}$ = effect of the interaction between season × hour, parity order × hour, days in milk × hour, or milk yield category × hour;
$\varepsilon_{ijk}$ = random error associated with $y_{ijk}$.

Data regarding daily mean MY and MF per cow were also compared among PO, DIM stage, and MYCat over the studied period using also a general linear model. A full factorial model was applied. The model is described below in Equation (2):

(2) $$y_{ijklm}=\mu +{PO}_{i}+{DIM}_{j}+{MYcat}_{k}+{\left(PO\times DIM\right)}_{ij}+{\left(PO\times MYCat\right)}_{ik}\;\;\;\;\;\;+{\left(DIM\times MYCat\right)}_{jk}+{\left(PO\times DIM\times MYCat\right)}_{ijk}+\delta_{l}+\varepsilon_{ijklm}$$

where

$y_{ijklm}$ = daily mean milk yield or milking frequency per cow;
μ = overall mean;
${PO}_{i}$ = effect of i-th level of parity order (i = 3);
${DIM}_{j}$ = effect of j-th level of days in milk (j = 3);
${MYCat}_{k}$ = effect of k-th level of milk yield category (k = 4);
${\left(PO\times DIM\right)}_{ij}$, ${\left(PO\times MYCat\right)}_{ik}$, ${\left(DIM\times MYCat\right)}_{jk}$, and ${\left(PO\times DIM\times MYCat\right)}_{ijk}$ = interaction term effects;
$\delta_{l}$ = random effect of l-th cow (l = 1 to 130);
$\varepsilon_{ijklm}$ = random error associated with $y_{ijklm}$.

In the models described above, the assumptions of residual homoscedasticity and normality were tested, and no violations were detected. Residual verification was performed using quantile–quantile plots, histograms, the Shapiro–Wilk test, and plots of residuals versus predicted values. Several variance–covariance matrices were tested, and the one with a lower corrected Akaike information criterion was selected. All statistical analyses were performed using SAS Institute Inc. (Version 3.81, Cary, NC, USA) [20]. Descriptive analyses were performed using SAS PROC MEANS. The general linear models were performed using SAS PROC GLIMMIX, while hourly MF was calculated using SAS PROC FREQ. Residual verifications were performed using SAS PROC UNIVARIATE and SAS PROC SGPLOT. Statistical significance was considered when *p* < 0.05. The Tukey–Kramer post hoc test was used when necessary.

## 3. Results

### 3.1. General Statistics of the Herd and AMS Performance

A median of 100 cows (interquartile range [IQR] = 22 cows) were milked per month across both AMS units during the study period, although this number varied by season: summer—median of 103 cows (IQR = 2 cows); autumn—median of 103 (IQR = 30 cows); winter—median of 81 cows (IQR = 3 cows); and spring—median of 100 cows (IQR = 8 cows). The median DIM for the herd was 164 days (IQR = 169 days), and cows had a median of two lactations (IQR = 1 lactation) during the studied period. The median milk yield per milking was 13.3 kg (IQR = 5.29 kg), while the median daily milk yield per cow was 38.0 kg (IQR = 11.51 kg). Each AMS performed a median of 135 milkings per day (IQR = 28.5 milkings) and a median of 5.5 milkings per hour (IQR = 3 milkings), resulting in an average of 11.6 ± 3.76% of cows being milked per hour. Cows were milked an average of 2.76 ± 0.73 times per day, with an average box time of 6.2 ± 1.6 min, which encompassed cow identification, teat preparation (pre-dipping, cleaning, and post-dipping), and milking. In general, 37% of the cows were milked ≤2 times daily, whereas 63% of the cows were milked ≥3 times daily during the studied period.

Higher MF was observed in primiparous cows (2.84 milkings/day; *p* < 0.0001), cows in early lactation (≤105 DIM, 3.00 milkings/day; *p* < 0.0001), and cows yielding ≥45 L/day (3.09 milkings/day; *p* < 0.0001) (Table 1). Higher daily milk yield was observed for cows in mid-lactation (106 to 205 DIM; 35.8 kg/day; *p* < 0.0001) and cows producing ≥45 kg/day (49.2 kg/day; *p* < 0.0001) (Table 1). We did not find differences for milk yield among PO (*p* = 0.1697) (Table 1).

### 3.2. Daily Patterns of Hourly Milking Frequency

**Figure 1:**
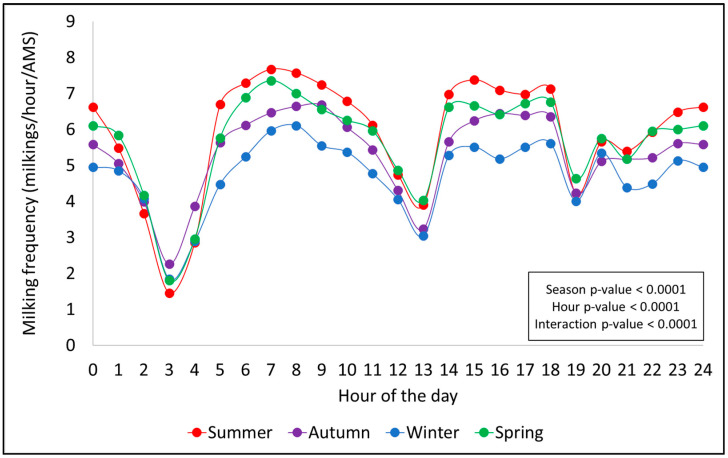
Hourly milking frequency distribution throughout the day for each season of the year.

**Figure 2:**
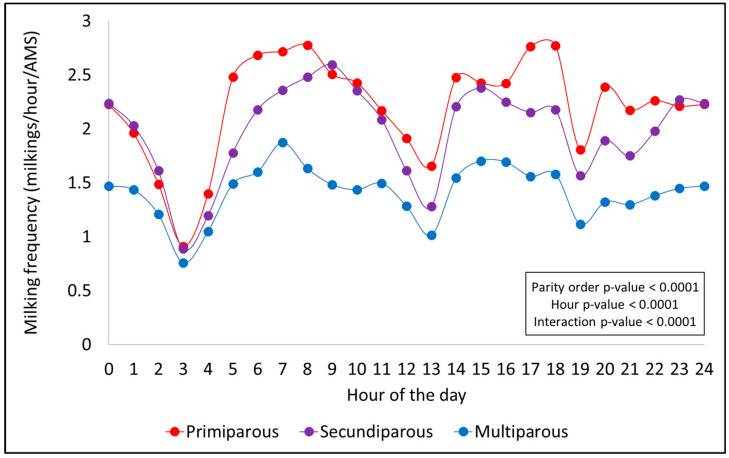
Hourly milking frequency distribution throughout the day for each parity order.

**Figure 3:**
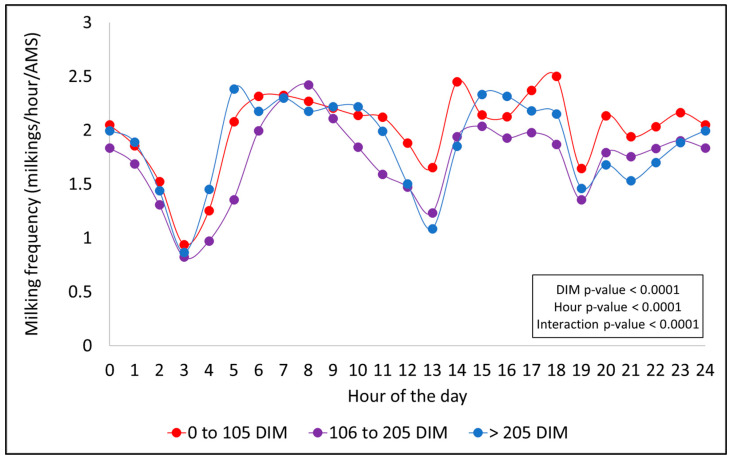
Hourly milking frequency distribution throughout the day for each stage of days in milk (DIM).

**Figure 4:**
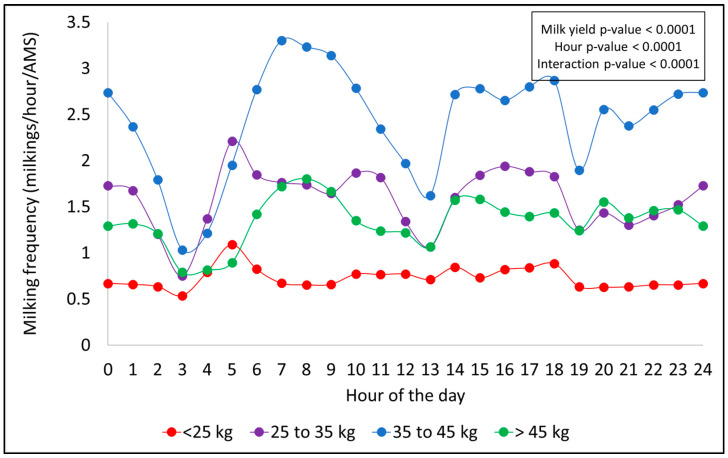
Hourly milking frequency distribution throughout the day for each milk yield category.

A consistent hourly milking pattern was observed throughout the day across all factors evaluated (season, PO, DIM, and MYCat), with peaks typically occurring during the early morning (5:00 a.m. to 11:00 a.m.) and in the afternoon periods (2:00 p.m. to 6:00 p.m.). Significant declines in MF were noted around 3:00 a.m. and between 7:00 and 8:00 p.m., coinciding with scheduled AMS cleaning times. In general, the magnitude and shape of these patterns varied according to each effect, as shown in Figure 1, Figure 2, Figure 3 and Figure 4.

Figure 1 presents the hourly distribution of milkings throughout the seasons. All the tested effects of season (*p* < 0.0001), hour (*p* < 0.0001), and their interaction (*p* < 0.0001) were significant. During the periods of highest milking activity (morning and afternoon), cows milked in summer and spring reached frequencies of 7 to 8 milking events per hour per AMS (Figure 1). In contrast, cows milked in autumn averaged 5 to 6 events, while those milked in winter showed the lowest activity, with 4 to 5 events per hour (Figure 1). This is likely a reflection of the reduced number of lactating cows during the colder months. However, when viewed overall, a consistent hourly milking pattern is evident throughout the seasons of the year, changing only in magnitude (Figure 1).

Figure 2 presents the hourly distribution of milkings for each PO. All the tested effects of PO (*p* < 0.0001), hour (*p* < 0.0001), and their interaction (*p* < 0.0001) were significant. Peaks in milking activity were also observed during morning and afternoon (Figure 2). Those peaks were considerably higher for primiparous cows, followed by secundiparous cows, compared to multiparous ones (Figure 2). This is likely due to a lower proportion of multiparous (26%) cows within the herd compared to primiparous (38%) and secundiparous cows (36%). However, overall, a consistent hourly milking pattern was evident, similar to what was observed across the seasons for the PO groups (Figure 2).

Figure 3 presents the hourly distribution of milkings for each DIM stage. All the tested effects of DIM stage (*p* < 0.0001), hour (*p* < 0.0001), and their interaction (*p* < 0.0001) were significant. A similar pattern observed for season and PO was found in the hourly MF across DIM stages, with peaks in milking activity occurring in the morning and afternoon (Figure 3). In addition, cows in early lactation (≤105 DIM) consistently showed a higher number of milkings, particularly during the nighttime period (from 7:00 p.m. to 12:00 a.m.) (Figure 3).

Figure 4 presents the hourly distribution of milkings for each MYcat. All the tested effects of MYcat (*p* < 0.0001), hour (*p* < 0.0001), and their interaction (*p* < 0.0001) were significant. In this case, the hourly milking pattern showed slight differences compared to the other evaluated factors. Both intermediate milk yield groups (25 to 35 and 35 to 45 kg/day) exhibited a similar pattern, with peaks in milking activity during morning and afternoon (Figure 4). For high-yielding cows (>45 kg/day), milking activity peaked later in the day, from 6:00 a.m. to 10:00 a.m., and remained elevated from 2:00 p.m. until 2:00 a.m. (Figure 4). Cows with a lower milk yield (<25 kg/day) showed a peak in milking activity at 5:00 a.m., followed by a relatively constant rate of approximately one milking every two hours per AMS over the day (Figure 4). This lower hourly MF is also associated with the smaller number of cows in this MY category throughout the year (only 19% of the cows with <25 kg/day) and milking permission settings based on milk yield.

## 4. Discussion

Our hypothesis that PO, DIM, and MYCat influence the daily temporal distribution of MF was confirmed. Regarding seasons, no clear seasonal effect on MF was observed, despite evidence that extended photoperiod and light intensity may enhance milk yield and composition [21]. In this respect, Hjalmarsson et al. [22] reported that reducing light intensity to 11 lx during nighttime does not affect cows’ general activity, such as gate passages and MF in barns with AMS. In a general way, in this dairy farm, the hourly MF pattern appeared to remain relatively constant throughout the year, being only slightly affected by those studied factors. It was observed that MF increased during the daytime and decreased during the nighttime and early morning hours. Milkings were concentrated in the morning (from 4 a.m. to 11 a.m.), as more cows have milking permission during this period. Given that milking permissions are set to allow most cows to be milked again within 5 to 7 h, a secondary peak in milking activity is typically observed in the afternoon (from 2:00 p.m. to 6:00 p.m.). The hourly MF pattern at this dairy farm is quite pronounced and aligns with findings in the literature, showing increased milking activity during the day and following feed delivery [15,16].

Management practices like feed delivery [9,10] and fetching cows [7,13] contribute to increased MF during specific hours of the day. Two feed deliveries were carried out on the farm, at 6:00 a.m. and 4:30 p.m., which appear to stimulate the morning milkings more effectively. This behavior is also influenced by the overnight fasting period, which increases hunger and stimulates feeding activity in the morning. As previously mentioned, this encourages cows to visit the milking units more frequently, thereby increasing MF around feeding activities.

On the other hand, the management of fetching cows seems to have an important effect on MF, as the times when the MF peaks correspond to when cows are fetched, especially around 5:00 a.m. and 1:00 p.m. at this farm. Cows requiring fetching are generally those experiencing health issues (such as lameness), those in the early stages of lactation (particularly primiparous animals), and those approaching the end of their lactation [13]. It can be observed that primiparous cows begin increasing their MF earlier in the morning compared to secundiparous and multiparous cows. A similar pattern can be observed in cows at the end of the lactation period (>205 DIM), as well as in cows with lower milk yield (<35 kg/day). A plausible explanation is that cows in these groups are fetched due to delayed voluntary visits, placing them late in the waiting queue, and as a result, they are milked earlier once fetched. In addition, if we consider a primiparous cow with a high milk yield (>35 kg/day) and relate them to the milking permission settings, these cows will generally receive milking permissions more frequently than others, resulting in a higher overall milking frequency.

Increasing voluntary milkings is a challenge in AMS. Nutritional strategies, including the quantity and physical form of concentrate offered, as well as the frequency of feed delivery at the bunk, may contribute to increased voluntary visits to the AMS [9,10,23]. While such practices are effective in promoting increased daily visits, a key challenge remains: “How to stimulate voluntary milking activity during nighttime periods?”. Based on our results, voluntary milking activity declines substantially during the early morning. This pattern appears to reflect inherent behavioral circadian rhythms in dairy cows, which may be challenging to alter through management interventions [16,22]. Although the use of automatic feeders or feed pushers may encourage more nighttime visits to the AMS, evidence suggests that excessive feed deliveries can disrupt natural cow behavior, leading to undesirable outcomes such as increased milking refusals and reduced lying time [21]. This supports the broader understanding that nighttime aligns with cows’ circadian rhythm and is primarily reserved for resting. Consequently, efforts to artificially increase voluntary milkings during this period are likely to be ineffective and may negatively impact both animal welfare and overall system performance. So, there is little we can do, since increasing voluntary night milkings is limited, and this natural behavior pattern should be accepted.

Finally, our study has some limitations, including the use of a single herd in the analysis. Factors related to farm management, genetics, and training cows to access the AMS, among others, are specific to this farm and may vary in others, even with similar or different cow traffic designs. Data on lame and sick cows (e.g., mastitis cases), which can influence MF, were not available. Information on refusals, which would be an interesting variable to explore, was only stored in individual cow files, which makes it challenging to integrate into a unified dataset. Nonetheless, refusals were likely minimal due to the farm’s guided cow traffic design. Furthermore, in this type of dataset, it is not possible to distinguish between milking events originating from a voluntary visit or from a fetched cow, as this would require video recordings or manual annotations, which are costly and labor-intensive to perform. Additionally, evaluations of MF in relation to DIM and MYCat are strongly influenced by the milking permission settings; however, these settings are standard in AMS systems. Additionally, social hierarchy likely can affect MF to some extent; however, classifying cows by rank and evaluating this factor is challenging, as cow interactions are limited to specific sub-groups at certain barn areas and moments throughout the day. Considering these limitations, our results are limited in generalization and should be interpreted with caution. On the other hand, we used data from a full year, encompassing 130 cows and all seasons. Future studies should focus on including multiple farms in their evaluations, as well as comparing different cow traffic designs, to determine whether the same MF pattern occurs in free versus guided traffic systems.

## 5. Conclusions

Hourly milking frequency in the studied AMS dairy farm followed a consistent daily pattern, with pronounced peaks in the morning (4:00 to 11:00 a.m.) and afternoon (2:00 to 6:00 p.m.), regardless of season, parity order, lactation stage, or milk yield. Although these factors significantly influenced overall milking frequency, their effect on the temporal distribution of milkings was limited. Cows in early lactation, primiparous cows, and high-producing cows were milked more frequently. Since this study was conducted on a single commercial farm, results may not be generalizable to herds with different management or traffic designs. Future multicenter studies are needed to validate these findings. Understanding these stable milking patterns can support optimization of cow traffic and labor management in AMS-equipped dairies.

## Figures and Tables

**Table 1:** Comparisons for milking frequency and milk yield among parity order, days in milk, and milk yield category for cows milked in an automatic milking system.

Variables	Number of Observations	Milking Frequency	SEM ^1^	*p*-Value	Milk Yield (L)	SEM	*p*-Value
Parity order				<0.0001			0.1697
Primiparous	13,315	2.84 a	0.019		34.94	0.096	
Secundiparous	12,524	2.67 b	0.011		35.13	0.056	
Multiparous	8410	2.68 b	0.016		35.14	0.081	
Days in milk (d)				<0.0001			<0.0001
≤105 d	11,124	3.00 a	0.008		34.46 c	0.039	
106 to 205 d	10,314	2.73 b	0.025		35.80 a	0.125	
>205 d	12,811	2.47 c	0.008		34.95 b	0.041	
Milk yield category				<0.0001			<0.0001
<25 kg	2567	2.52 c	0.034		20.28 d	0.170	
≥25 to <35 kg	9993	2.54 c	0.008		30.98 c	0.038	
≥35 to <45 kg	14,895	2.79 b	0.006		39.83 b	0.029	
≥45 kg	6794	3.09 a	0.010		49.20 a	0.047	
Parity order × Days in milk				<0.0001			<0.0001
Parity order × Milk yield category				0.0111			<0.0001
Days in milk × Milk yield category				<0.0001			<0.0001
Parity order × Days in milk × Milk yield category				<0.0001			<0.0001

^1^ Standard error of the mean. Different letters in the columns indicate statistically significant differences considering the Tukey–Kramer post hoc test.

## Data Availability

Data is contained within the article.

## Abbreviations

The following abbreviations are used in this manuscript:

AMS	Automatic milking system
DIM	Days in milk
IQR	Interquartile range
MF	Milking frequency
MYCat	Milk yield category
PO	Parity order
